# GP involvement after a cancer diagnosis; patients’ call to improve decision support

**DOI:** 10.3399/bjgpopen20X101124

**Published:** 2020-12-09

**Authors:** Eveline A Noteboom, Ietje AA Perfors, Anne M May, Mariken E Stegmann, Saskia FA Duijts, Ella A Visserman, Vivian Engelen, Carol Richel, Elsken van der Wall, Niek de Wit, Charles W Helsper

**Affiliations:** 1 Julius Center for Health Sciences and Primary Care, University Medical Centre Utrecht, Utrecht, the Netherlands; 2 Department of General Practice and Elderly Care Medicine, University Medical Center Groningen, Groningen, the Netherlands; 3 Netherlands Comprehensive Cancer Organisation, Utrecht, the Netherlands; 4 Public and Occupational Health, Amsterdam UMC, Amsterdam, the Netherlands; 5 Dutch Federation of Cancer Patient Organisations, Utrecht, the Netherlands; 6 Dutch Breast Cancer Association, Utrecht, the Netherlands; 7 Department of Medical Oncology, University Medical Centre Utrecht, Utrecht, the Netherlands

**Keywords:** neoplasms, general practice, decision making, shared

## Abstract

**Background:**

Shared decision making (SDM) is considered important to realise personalised cancer care. Increased GP involvement after a diagnosis is advocated to improve SDM.

**Aim:**

To explore whether patients with cancer are in need of GP involvement in cancer care in general and in SDM, and whether GP involvement occurs.

**Design & setting:**

An online national survey was distributed by the Dutch Federation of Cancer Patient Organisations (NFK) in May 2019.

**Method:**

The survey was sent to (former) patients with cancer. Topics included GP involvement in cancer care in general and in SDM. Descriptive statistics and quotes were used.

**Results:**

Among 4763 (former) patients with cancer, 59% (*n* = 2804) expressed a need for GP involvement in cancer care. Of these patients, 79% (*n* = 2193) experienced GP involvement. Regarding GP involvement in SDM, 82% of patients (*n* = 3724) expressed that the GP should 'listen to patients' worries and considerations', 69% (*n* = 3130) to 'check patients' understanding of information', 66% (*n* = 3006) to 'discuss patients' priorities in life and the consequences of treatment options for these priorities', and 67% (*n* = 3045) to 'create awareness of the patient’s role in the decision making'. This happened in 47%, 17%, 15% and 10% of these patients, respectively.

**Conclusion:**

The majority of (former) patients with cancer expressed a need for active GP involvement in cancer care. GP support in the fundamental SDM steps is presently insufficient. Therefore, GPs should be made aware of these needs and enabled to support their patients with cancer in SDM.

## How this fits in

Little is known about patients’ needs for GP involvement in cancer care and in shared decision making (SDM), and to what extent GP involvement occurs. This study showed that the majority of (former) patients with cancer had a need for GP involvement in cancer care and in SDM. However, GP involvement in SDM was infrequently experienced. Therefore, GPs should be made aware of these needs and enabled to support their patients to make personalised cancer treatment decisions.

## Introduction

Cancer treatment decisions have become more complex, owing to the increasing number of treatment options. This enables a more personalised approach.^1^ Incorporating personal preferences in treatment decisions requires SDM. SDM aims to establish a treatment decision that optimally matches a patient’s personal preferences and expectations.^2^ An effective SDM process consists of four steps: 1) awareness of choice; 2) explanation of treatment options; 3) time for deliberation; and 4) making an informed decision.^2^

Unfortunately, in the present hospital-oriented cancer care pathway, essential steps for successful SDM are usually insufficiently supported. First, patients with cancer are often unaware of their important role in choosing the ‘best-fitting’ treatment.^3^ Second, medical information, including treatment options, is often not understood by patients with cancer.^4^ Third, time for deliberation is often limited, since the short in-hospital pathway between diagnosis and treatment choice generally does not facilitate reflection. This leaves little room to consider treatment options in the light of patients' personal preferences and expectations.^3,5,6^

GPs usually have longstanding relationships with their patients. Consequently, for many, the GP is the ‘trusted healthcare professional’, with longitudinal knowledge of their patients’ medical and personal history.^1,7^ Hence, the GP is considered to be in the ideal position to guide the patient through the different steps of the SDM process.^1,6^ Patients with cancer and GPs support this extended role for the GP in cancer treatment decision making; for example, through determining patients' preferences, discussing treatment options, and explaining medical information.^8–10^

Positive effects of increased GP involvement after a cancer diagnosis have been described previously. Wallner *et al* showed that a patient’s experience of GP engagement, that is, how informed the patient felt the GP was about the diagnosis, was associated with higher satisfaction of treatment decisions in cancer.^11^ Wieldraaijer *et al* showed that a consultation with the GP between diagnosis and start of treatment is beneficial for patients' feelings of comfort and satisfaction.^12^ It has been demonstrated earlier by the authors of the present study that a cancer-related GP consultation before treatment decision may improve the SDM process of palliatively treated patients with cancer, according to patients, GPs, and treating physicians.^13^

Despite this broadly shared call for more GP involvement in the process of making cancer treatment decisions, little is known about patients’ perspectives. Therefore, the study aimed to explore patients’ needs for GP involvement after a cancer diagnosis in general and in SDM, and whether this GP involvement occurred.

## Method

### Design

An online national survey was developed and distributed among (former) patients with cancer in the Netherlands in May 2019 by the NFK.

### Study population

NFK is an umbrella organisation of 19 cancer patient organisations. These organisations together represent approximately 35 000 (former) patients with cancer. The survey was distributed in several ways. First, the survey was dispersed to the affiliated cancer patient organisations, which represent adult patients with a large variety in diagnoses. These organisations were asked to distribute the survey among their members. This could either be directly to all members or indirectly through their newsletter. Second, a web link to the survey was distributed through social media accounts of NFK (Facebook, LinkedIn, Twitter, and Instagram), via their website, and via other relevant partner organisations (such as The Dutch Cancer Society and the website kanker.nl). Finally, a panel of (former) patients with cancer, who were not members of one of the cancer patient organisations, were sent invitations to participate in the survey. These patients registered voluntarily to receive invitations for NFK surveys and were not selected for this specific survey.

### Online survey

The online survey was developed by NFK, in cooperation with experts in the fields of cancer, primary care, and SDM, including patients, clinicians, researchers, and policymakers. The survey consisted of the following two parts: one part focused on the role of the GP; and the other on the role of the specialised oncology nurse. This study only used data from the GP-related questions.

The survey started with a selection question, only participants who responded yes to the question, ‘Do, or did, you have cancer?’ were able to proceed with filling in the questionnaire. Then, eight general questions about patient and disease characteristics followed. Hereafter, 10 questions addressing the patient’s personal needs for GP involvement in cancer care were posed. These questions covered the following topics: 1) the need for GP involvement in cancer care at any time after diagnosis; 2) whether this GP involvement occurred; 3) the need to have SDM topics addressed in a GP consultation; and 4) whether these topics were actually addressed. Finally, the survey assessed 5) the initiator of involvement of the GP in cancer care and 6) satisfaction with GP involvement in cancer care (see Supplementary Appendix S1 for the survey).

GP involvement in cancer care was defined as: 'Any type of long or short contact with the GP about the diagnosis, treatment and/or its consequences. This could either be via telephone, an appointment at the GP’s office, or a home-visit.' The SDM topics included that the GP should: 1) 'Listen to my worries and considerations about the diagnosis, treatment and its consequences'; 2) 'Check if I understand the information about my diagnosis, treatment and its consequences'; 3) 'Discuss what I think is important in my life and the consequences of treatment options for these priorities'; and 4) 'Explain to me the importance of my own opinion when making a treatment decision'.

The format of the questions was either closed (numeric, multiple choice) or open-ended. Needs and the occurrence of GP involvement were assessed with multiple-choice questions and open-ended questions for clarification. Satisfaction with GP involvement in cancer care was scored on a 10-point number rating scale ranging from 1 (very unsatisfied) to 10 (very satisfied). The estimated time to complete the questionnaire was approximately 5–10 minutes. The data were collected with the online tool SurveyMonkey. Responders participated anonymously in the survey. The survey was open for response for 2 weeks. Responders could choose to answer only part of the questions. Only if the general questions and the question, ‘Did you have a need for contact with your GP about your cancer diagnosis, the treatment and/or its consequences?’ were answered with ‘yes’, ‘no’ or ‘don’t know/NA’, the survey was used in the analysis.

### Analysis

Descriptive analyses of the closed questions were performed for the total population and for subgroups of the following characteristics: sex, age, education, type of cancer, cancer stage, and time since last treatment. Statistical testing was not performed, since with the current number of patients, small often not (clinically) relevant differences would already be statistically significant. Categorical variables are presented as numbers and percentages. Continuous variables are presented, depending on whether or not normally distributed, with means and standard deviations (SD) or medians and interquartile ranges (IQR). All analyses were performed with IBM SPSS Statistics (version 25). Relevant quotes from the open questions were used to illustrate the results.

## Results

### Patient characteristics

The survey was completed by 4763 (former) patients with cancer. The mean age of responders was 62 years (SD ±12), 56% were female, and 48% of the responders had a high education level (Table 1). The majority of the responders were diagnosed with either breast cancer (26%), haematological cancers (18%), or colorectal cancer (16%). The median time since the last received cancer treatment was 2 years (IQR 1–6) and 46% reported to be cured.

**Table 1.** Baseline characteristics of responders

	Total, *N* = 4763
	*n*	*%*
**Female**	2686	56
**Age**, years, mean (± SD)	62	(±12)
**Education^a^**		
High	2276	48
Middle	1908	40
Low	464	10
Other	61	1
Missing	54	1
**Diagnosis**		
Breast cancer	1231	26
Haematological cancers	874	18
Colorectal cancer	787	16
Prostate cancer	569	12
Bladder cancer	270	6
Gynaecologic cancer	179	4
Lung cancer	153	3
Melanoma	125	3
Esophageal cancer	105	2
Other	470	10
**Years since last received cancer treatment**, median (IQR)	2	1–6
**Patient reported cancer stage**		
Cured	2166	46
Will probably be cured	901	19
Will probably not be cured	1256	26
Don’t know/NA	440	9

^a^Education is categorised as high (university or higher professional education), middle (secondary education), and low (primary education or no education). IQR = interquartile range. NA = not applicable. SD = standard deviation.

### GP involvement in general

Of all responders, 59% (*n* = 2804) expressed a need for GP involvement in cancer care any time after diagnosis (Table 2). GP involvement in cancer care was experienced by 79% (*n* = 2193) of these responders. A relatively high need for GP involvement was reported by females (female: 64%; male: 52%). GP involvement occurred more often in males (82%) than in females (77%). A relatively high need for GP involvement was reported by patients with lung, oesophageal, and gynaecologic cancer (68%–69%), versus other cancers (47%–64%). A relatively small proportion of (former) patients with breast and gynaecologic cancer experienced GP involvement (74%–76%), compared with other cancers (78%–88%). Responders who indicated 'will probably not be cured' reported relatively high need of GP involvement (66%) compared with those who indicated to be 'cured' (55%). The latter group reported GP involvement less often (75% versus 85%). Quotes in Supplementary Box S1 illustrate the need for, and lack of, experiences with GP involvement in cancer care.

**Table 2.** Need for GP involvement in cancer care and whether GP involvement occurred. Presented for total and stratified per subgroup

Need for GP involvement in cancer care any time after diagnosis
	Need (yes)	Contact occurred? (yes)^a^
	Total	Of total	Of need
	*n*	*n*	%	*n*	%
All responders	4763	2804	59	2193	79
**Sex**					
Male	2077	1073	52	873	82
Female	2686	1731	64	1320	77
**Age**					
Aged <65 years	2537	1577	62	1245	80
Aged ≥65 years	2226	1227	55	948	78
**Education**					
Low education^b^	464	254	55	188	75
Middle education^b^	1908	1134	59	849	76
High education^b^	2276	1351	59	1105	82
**Diagnosis**					
Haematological cancers	874	478	55	380	80
Colorectal cancer	787	402	51	307	78
Bladder cancer	270	128	47	105	83
Gynaecologic cancer	179	121	68	91	76
Melanoma cancer	125	75	60	64	85
Breast cancer	1231	791	64	582	74
Prostate cancer	569	323	57	276	86
Lung cancer	153	105	69	83	81
Oesophageal cancer	105	72	69	63	88
**Years since last received cancer treatment**					
Last treatment ≤2 years ago	2404	1462	61	1215	84
Last treatment ≥3 years ago	2359	1342	57	978	74
**Patient reported cancer stage**					
Cured	2166	1180	55	875	75
Will probably be cured	901	535	59	413	78
Will probably not be cured	1256	825	66	699	85

^a^Percentage ‘Contact occurred? (yes)’ is calculated for those who responded to have a need for GP involvement and filled in the question ‘Contact occurred?’. Denominators vary for this question and are slightly lower than the total number of patients who indicated to have a need for GP involvement, due to missing data (i.e. not all respondents filled in the follow-up question ‘Contact occurred’). Further information is available from the authors on request ^b^Education is categorised as high (university or higher professional education), middle (secondary education), and low (primary education or no education).

### GP involvement in SDM

Table 3 shows the needs to have SDM topics addressed in a GP consultation and whether these topics were actually addressed. Eighty-two per cent (*n* = 3724) of the responders expressed that their GP should listen to their worries and considerations about the diagnosis, treatment, and its consequences. This actually happened in 47% (*n* = 1744) of these cases. The majority of the responders expressed that the GP should: 'check understanding of information' 69% (*n* = 3130); 'discuss patients' priorities in life and the consequences of treatment options for these priorities' 66% (*n* = 3006); and 'explain importance of patients' opinions in decisions' 67% (*n* = 3045). These topics were addressed in respectively 17% (*n* = 542), 15% (*n* = 461), and 10% (*n* = 294) of these cases.

**Table 3.** Needs to have important topics in the shared decision-making process for cancer treatment addressed in a GP consultation and whether this topic was addressed

My GP should …	Listen to my worries and considerations about the diagnosis, treatment and its consequences	Check if I understand the information about my diagnosis, treatment, and its consequences	Discuss what I think is important in my life and the consequences of treatment options for these priorities	Explain to me the importance of my own opinion when making a treatment decision
Need (yes)	**Topic addressed?** **(yes)** ^**a**^	Need (yes)	Topic addressed? (yes)^a^	Need (yes)	Topic addressed? (yes)^a^	Need (yes)	Topic addressed? (yes)^a^
	Total	Of total	Of need	Of total	Of need	Of total	Of need	Of total	Of need
	*n*	*n*	%	*n*	%	*n*	%	*n*	%	*n*	%	*n*	%	*n*	%	*n*	%
**All responde** **r** **s**	4526	3724	82	1744	47	3130	69	542	17	3006	66	461	15	3045	67	294	10
**Sex**																	
Male	1966	1561	79	755	48	1349	69	236	18	1274	65	217	17	1297	66	128	10
Female	2560	2163	85	989	46	1781	70	306	17	1732	68	244	14	1748	68	166	10
**Age**																	
Aged <65 years	2434	2059	85	1008	49	1685	69	326	19	1639	67	257	16	1665	68	160	10
Aged ≥65 years	2092	1665	80	736	44	1445	69	216	15	1376	65	204	15	1380	66	134	10
**Education**																	
Low education^b^	422	326	77	127	39	308	73	51	17	296	70	33	11	296	70	32	11
Middle education^b^	1810	1495	83	644	43	1305	72	221	17	1237	68	183	15	1239	69	119	10
High education^b^	2185	1812	83	930	51	1439	66	260	18	1394	64	238	17	1435	66	138	10
**Diagnosis**																	
Haematological cancers	832	680	82	311	46	530	64	90	17	512	62	75	15	507	61	38	8
Colorectal cancer	732	568	78	254	45	517	71	94	18	469	64	65	14	483	66	42	9
Bladder cancer	256	201	79	83	41	176	69	26	15	176	69	14	8	176	69	10	6
Gynaecologic cancer	170	141	83	66	47	119	70	16	13	119	70	17	14	127	75	11	9
Melanoma cancer	119	97	82	51	53	87	73	13	15	79	66	18	23	83	70	11	13
Breast cancer	1178	1002	85	442	44	818	69	143	18	801	68	104	13	804	68	76	10
Prostate cancer	543	447	82	229	51	384	71	70	18	358	66	67	19	377	69	52	14
Lung cancer	145	124	86	64	52	104	72	20	19	105	72	26	25	95	66	12	13
Oesophageal cancer	104	84	81	54	64	76	73	15	20	75	72	19	25	78	75	12	15
**Years since last received cancer treatment**																	
Last treatment ≤2 years ago	2307	1897	82	995	53	1532	66	289	19	1510	66	289	19	1497	65	171	11
Last treatment ≥3 years ago	2219	1827	82	749	41	1598	72	253	16	1496	67	172	12	1548	70	123	8
**Patient reported cancer stage**																	
Cured	2035	1658	82	711	43	1438	71	228	16	1340	66	147	11	1398	69	99	7
Will probably be cured	867	708	82	329	47	606	70	116	19	570	66	81	14	598	69	52	9
Will probably not be cured	1208	1017	84	553	54	806	67	142	18	818	68	200	24	776	64	115	15

^a^Percentage ‘Topic addressed? (yes)’ is calculated for those who responded to have a need for GP involvement and filled in the question ‘Topic addressed?’ Denominators vary for this question and are slightly lower than thetotal number of patients who indicated to have a need to have SDM topics addressed by the GP, dueto missing data (i.e. not all respondents filled in the follow-up question‘Topic addressed’). Further information is available from the authors onrequest. ^b^Education is categorised as high (university or higher professional education), middle (secondary education), and low (primary education or no education).

In all subgroups, the need for GP involvement in the SDM process was high. However, this GP involvement in SDM was infrequently experienced by responders, especially by responders aged ≥65 years, those with low education, those with breast, bladder, gynaecologic, haematological cancers, or colon cancer, and by the 'cured' group of responders. Quotes that illustrate the need for GP involvement in SDM are presented in Supplementary Box S1.

### Initiator and satisfaction

Among those who reported that their GP was involved in cancer care, this was initiated by the patient in 52% (*n* = 1650), by the GP in 31% (*n* = 987), by family and friends in 4% (*n* = 116), and unknown in 13% (*n* = 421) (data not shown). In case of GP involvement, satisfaction with GP involvement in cancer care was evaluated with a mean score of 7.4 (±2.4). This involvement was rated higher if the GP was the initiator (8.0±2.0), instead of the patient (7.0±2.4). This is illustrated by the final quote in Supplementary Box S1.

## Discussion

### Summary

In the present study, the needs of (former) patients with cancer were evaluated for GP involvement in cancer care. More than half of the responders reported that they wanted the GP to be involved in cancer care after the diagnosis. GP involvement in cancer care was experienced in over three-quarters of these cases. As for GP involvement in SDM for cancer treatment, the balance is different. Although more than 80% expressed a need for the GP to listen to worries and considerations, this support was lacking in just over half of these cases. Also, more than two-thirds of responding patients with cancer indicated a need to have elemental SDM topics addressed in a GP consultation, such as explaining information, checking understanding, and discussing priorities. This SDM support was only experienced in a small minority of cases. Finally, the initiator of GP involvement was mostly the patient, whereas satisfaction with GP involvement in cancer care was higher if the GP was the initiator.

### Strengths and limitations

This study has several limitations. First of all, recall bias may have occurred, since the median interval between last received treatment and participation was 2 years. Among those treated longer ago (≥3 years) the reported needs were similar to those treated ≤2 years ago. However, those treated ≥3 years ago reported GP involvement less often. This could be the result of an underestimation of the actual GP involvement, owing to incorrect recall. Second, the network used to recruit patients with cancer may have addressed a selective population. The survey was distributed among a group of (former) patients with cancer who are in some way affiliated to a cancer patient organisation. Consequently, the responders may have been relatively committed, active, and critical, thus may have different needs than the average patient with cancer and have a stronger drive to meet those needs. Within this population, selective response may have occurred, as those being very satisfied or unsatisfied with GP involvement may be more inclined to participate in a survey about corresponding needs. Selective participation is supported by the relatively high percentage of patients with a high education (48%). However, the percentage of females (56%) and the average age (62 years) in the sample is comparable with the Dutch population of patients with cancer.^14^

The main strength of this study is the high number of (former) patients with cancer who responded to this survey. The large population and the variety of cancer types support generalisability and enabled subgroup explorations.

### Comparison with existing literature

To the authors' knowledge, this is the first study among (former) patients with cancer that combines an exploration of the needs for GP involvement in cancer care and specifically in SDM, and to what extent GP involvement occurred. The findings are in line with the few studies that have addressed adjacent topics. It confirms the need for a supportive role of the GP as previously demonstrated.^10,15^ It also confirms the conclusion of Halkett *et al*, who reported that patients with cancer see a role for the GP in SDM support after a cancer diagnosis.^10^ Lang *et al* reported that 34.5% of the patients with cancer discussed diagnostic and therapy-related decisions with the GP.^9^ Also, Klabunde *et al* showed that 64.2% of the GPs reported to explore patients' preferences for treatment.^8^ Both percentages are higher compared with the 15% of the (former) patients with cancer who reported SDM involvement by the GP in the present study. This might be owing to a different study population or because of differences in perception between GPs and patients of what is actually addressed during the consultation. Additionally, the results imply that GPs generally provide supportive care, mostly including the discussion of worries and considerations, but the discussion of the cancer treatment decision itself is often lacking. This might be caused by GPs’ unawareness of patients’ needs for SDM support, or by reluctance among GPs because of perceived lack of expertise.^13,16^

Furthermore, the results show that satisfaction with GP involvement is scored higher if the GP is the initiator of contact. This is supported by findings in a qualitative study by Brandenbarg *et al* among curatively treated patients with colorectal cancer who expressed dislike when the GP did not initiate contact after treatment.^15^ Also, cancer patients’ preferences for initiation of contact by the GP is expressed for other conversations, such as for advanced care planning.^17^ In addition, previous studies show that patients with cancer are more satisfied if the GP is informed about the diagnosis^11^ and if there is a contact moment with the GP (a 'time-out consultation') before the start of treatment.^12^ The findings also support and explain the potential positive effect on SDM of actively involving the GP between diagnosis and therapy choice, which was recently reported for palliatively treated patients with cancer.^13^

### Implications for practice

Treating physicians and GPs should actively explore patients’ needs for GP involvement after a cancer diagnosis, particularly for SDM. GPs should be aware that patients wish to have cancer treatment decision-related topics addressed by the GP. GP support could be enabled to support SDM in the hospital, for instance, by actively offering a 'time-out consultation' with the GP with SDM tools.^12,13,18,19^ In addition, cancer patient organisations could support GP involvement by empowering patients to discuss preferred topics with their GP.

In conclusion, even though patients experience GP support after a cancer diagnosis, their needs for support in the SDM process often remain unanswered. GPs can do better in checking understanding of information, discussing patients’ priorities and preferences, and explaining the importance of patients’ own opinions in decision making. Since GPs seem adequately equipped to provide the desired SDM support, GPs and hospitals should join forces to make sure that GPs can and will support their patients when faced with important medical decisions.
